# A Case of Myopericarditis and Cardiac Tamponade as the Initial Presentation of COVID-19 Infection

**DOI:** 10.7759/cureus.12967

**Published:** 2021-01-28

**Authors:** Michael A Gioia, Adeniyi Okunade, Adam Friedman, Muhammad F Ahmed, Rumman A Syed

**Affiliations:** 1 Internal Medicine, Brookdale University Hospital Medical Center, Brooklyn, USA

**Keywords:** covid-19, tamponade, pericardial effusion, myocarditis, covid-induced myocarditis

## Abstract

Coronavirus disease 2019 (COVID-19) is a novel disease with various complications involving different organ systems caused by the severe acute respiratory syndrome coronavirus 2 (SARS-CoV-2) virus. While the respiratory complications associated with COVID-19 have been well publicized, our understanding of the nonpulmonary complications of COVID-19 is lacking. Herein we present a case of a middle-aged woman who developed myopericarditis, pericardial effusion, and tamponade in the setting of COVID-19 infection.

## Introduction

Myopericarditis is a condition in which the myocardium and pericardium of the heart is inflamed, resulting in reduced cardiac function, pericardial effusion and can lead to cardiac tamponade in some cases if not promptly treated. Viruses are the most common cause of myocarditis and pericarditis in the developed world [1]. Among the viral causes of myopericarditis, adenoviruses and enteroviruses are the most prevalent [2]. While the prevalence of viral myocarditis/pericarditis is well established, little is known about the cardiac complications of coronaviruses. Previous coronavirus outbreaks, mainly the severe acute respiratory syndrome coronavirus (SARS-CoV) (2002-2004) and Middle East respiratory syndrome coronavirus (MERS-CoV) (2012-2013) also caused severe respiratory illnesses, similar to the current pandemic SARS-CoV-2; however, there is a scarcity of published evidence of direct cardiac involvement from these previous coronavirus outbreaks. So far, there are only a few published cases of direct cardiac involvement [3-5] in coronavirus disease 2019 (COVID-19) and we do not yet know the full extent of cardiac involvement in SARS-CoV-2 infection.

## Case presentation

**Figure 1:**
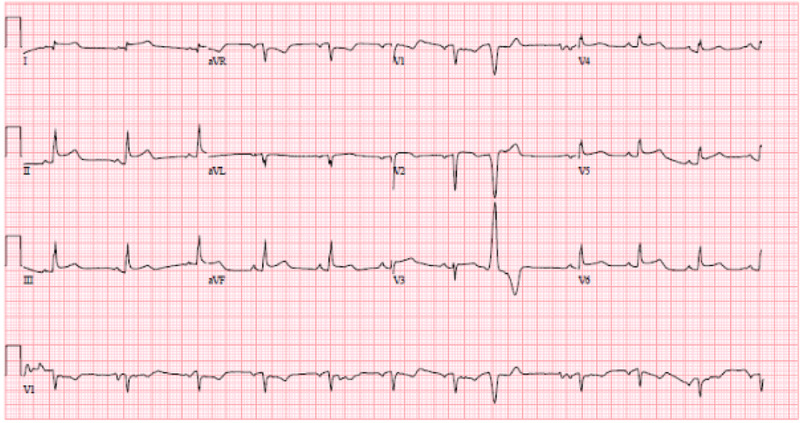
EKG on admission. EKG, electrocardiogram

**Figure 2:**
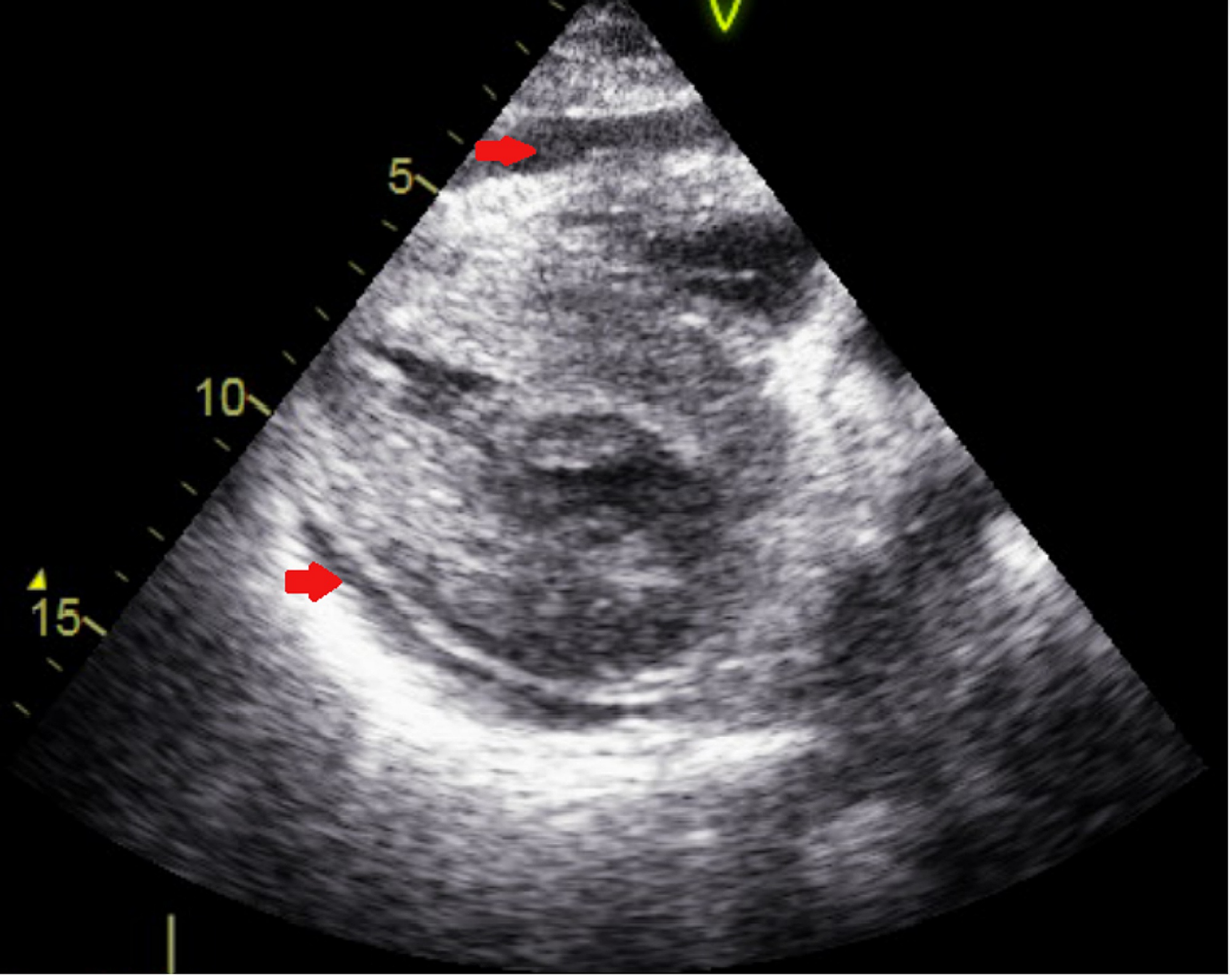
Initial echocardiogram showing pericardial effusion. Red arrows: pericardial effusion

**Figure 3:**
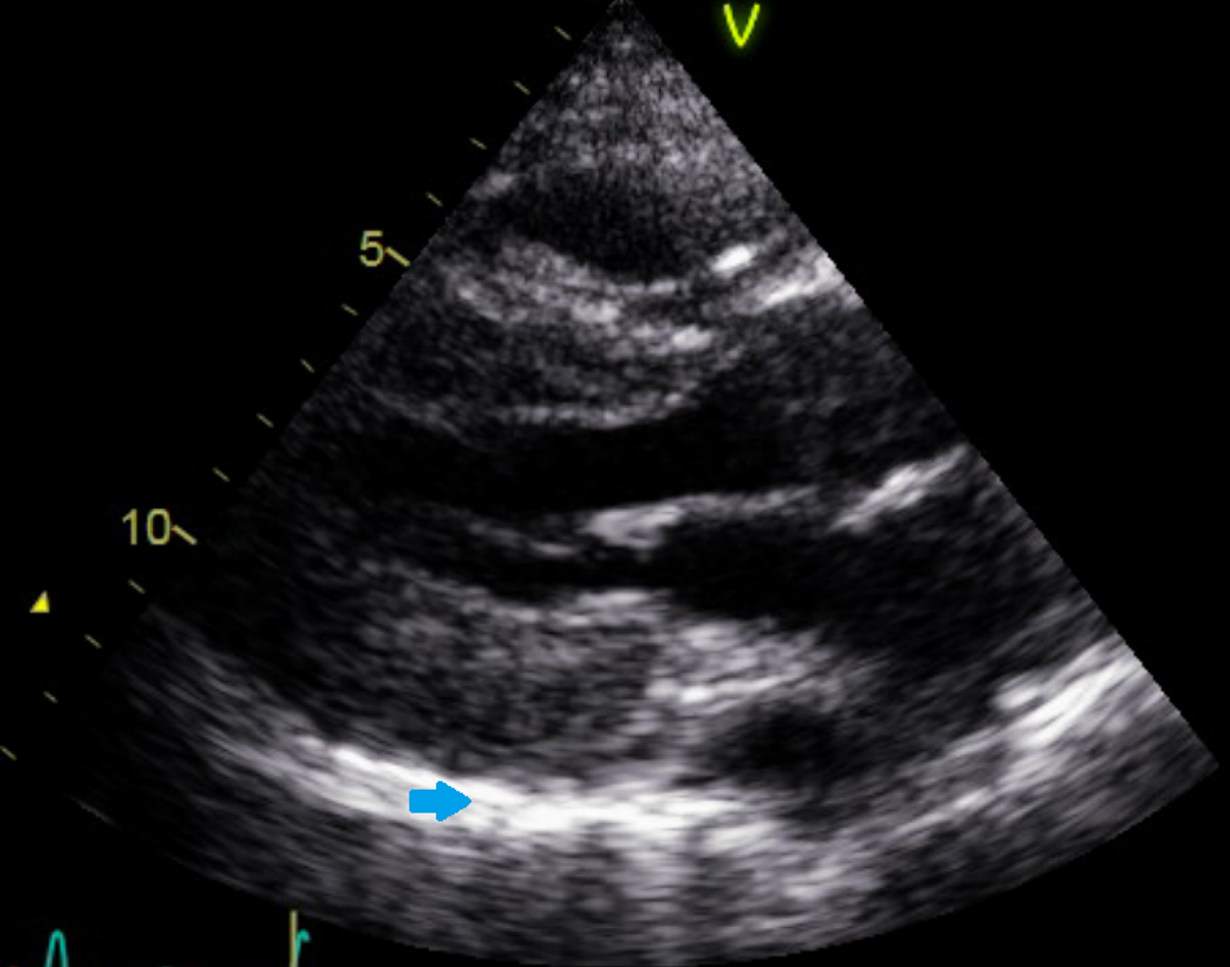
Echocardiogram after pericardiocentesis. Blue arrow: indicating pericardium, now with resolution of the previously seen effusion.

A 57-year-old female with a past medical history of hypertension and active tobacco smoking presented with a chief complaint of trouble breathing. She was unable to provide further history due to severe respiratory distress. Initial vitals were: temperature 36.3 degree celsius, heart rate 106 beats per minute, respiratory rate 30 breaths per minute, blood pressure 98/58 mmHg, and oxygen saturation was 90% on nonrebreather mask at 15 liters per minute. Electrocardiogram (EKG) was performed and showed diffuse ST segment elevations, and her initial troponin (intact) level was noted to be 64.0 ng/mL. Transfer was then arranged to the nearest percutaneous coronary intervention (PCI) center, Brookdale University Hospital Medical Center (BUHMC). Prior to transport, the patient had cardiac arrest x2 due to hypoxic respiratory failure and was intubated and started on vasopressors. The patient was then transferred. EKG repeated on arrival to BUHMC was essentially unchanged from the previous one (Figure 1). COVID-19 nasopharyngeal polymerase chain reaction (PCR) test was sent. Within a few minutes of arrival, the patient had another cardiac arrest with initial rhythm of pulseless electrical activity (rhythm on the monitor was sinus bradycardia). Return of spontaneous circulation was achieved after four minutes and the patient was taken emergently to the cath lab. Bedside echocardiogram done prior to coronary angiography showed significant hypertrophy of the left ventricle and moderate pericardial effusion (Figure 2). Left heart catheterization showed nonocclusive coronary artery disease (CAD) with preserved ejection fraction (EF). Right heart catheterization found equalization of right heart pressures (pulmonary capillary wedge pressure 17 mmHg, right ventricular end diastolic pressure 18 mmHg, right atrial end diastolic pressure 17 mmHg). Emergent pericardiocentesis was performed with immediate drainage of 460 cc serous fluid. After pericardiocentesis, pulmonary capillary wedge pressure was 23 mmHg and right atrial diastolic pressure was 12 mmHg. She was then taken back to coronary care unit (CCU) and started on targeted temperature management (TTM), and at that time was started on norepinephrine at a rate of 30 mcg/min and vasopressin at a rate of 0.03 units/min. Chest X-ray done showed mild pulmonary congestion. Laboratory findings were consistent with acute renal failure (blood urea nitrogen, BUN/Creatinine 93/3.37 from baseline 13/0.90 six months prior), shock liver (alanine transaminase, ALT >2000, aspartate aminotransferase, AST >1500), high anion gap metabolic acidosis (pH 7.05, anion gap 29). COVID-19 nasopharyngeal PCR test came back positive. Official echocardiogram was done which showed severely reduced EF (15%-25%) and diffuse hypokinesis of the left ventricular wall, pericardial effusion resolved with drain in place (Figure 3). Throughout the day, the patient's vasopressor requirement increased. She then became bradycardic (sinus bradycardia), which did not respond to 1 mg of atropine so TTM stopped and the patient was rewarmed. Repeat blood work did not show any significant improvement in acidosis, renal or hepatic function. Heart rate dropped to the 30s so the patient was started on transcutaneous pacing. Arterial line inserted as blood pressure cuff was not able to consistently assess blood pressure and arterial blood gas sent. Arterial blood gas showed severe metabolic acidosis (pH 6.90, bicarbonate 3.5) with respiratory alkalosis (PCO2 18.5). Arterial line lost waveform shortly after being placed, and the patient had no palpable pulse. Transcutaneous pacemaker temporarily turned off to assess intrinsic heart rhythm and found asystole. Further resuscitative efforts deemed to be medically futile by the treating team and the patient was pronounced deceased.

## Discussion

During previous coronavirus and influenza epidemics, significant cardiovascular complications of these viruses have been well documented [6]. The mechanisms by which the novel coronavirus causes cardiac injury is not surprising; what is intriguing is whether SARS-CoV 2 is more virulent on the cardiovascular system than other viruses [6]. Since the onset of the current COVID-19 pandemic, there have been reports of (extra)pulmonary manifestations, however, there have been very few documented cases of pericardial involvement [3].

One of the early studies from Wuhan, China among 41 hospitalized patients revealed five (12%) had acute cardiac injury (as defined as one of: high-sensitivity troponin-I level > the 99th percentile, new abnormalities on EKG or new wall-motion abnormalities on echocardiogram), out of which four patients required intensive care. These patients, as we found in our patient, manifested increased high-sensitivity cardiac troponin I, abnormalities on EKGs, and heart ultrasound [7]. In a similar study of 138 patients, Wang et al. also found 7.2% had acute cardiac injury, with most patients requiring intensive care [8].

The significance of cardiac injury in COVID-19 mortality was also reported by Shi et al. in a study of 416 patients hospitalized with COVID-19, of whom 57 died. Approximately 20% of patients had acute cardiac injury defined as high-sensitivity troponin-I greater than the 99% percentile upper reference limit. These patients also had a higher incidence of acute respiratory distress syndrome (ARDS) (58.5% vs 14.7%; p < 0.001) and a higher mortality rate (51.2% vs 4.5%; p < 0.001) than those without cardiac injury [9].

It has been suggested that viruses cause pericardial inflammation by inflammatory response and/ or direct cytotoxic effects [3]. COVID-19 have also been reported to trigger an exaggerated systemic inflammatory response, with patients requiring ICU care having higher levels of pro-inflammatory cytokines, suggesting that cytokine storm has some correlation with disease severity [7]. Some COVID-19 patients were also noted to have significant levels of anti-inflammatory cytokines, suggesting that more studies are needed to explain the pathogenesis [7]. 

## Conclusions

Respiratory system involvement in COVID-19 infection has been well established, however, as the global pandemic continues, we have seen the infection’s effects on other organ systems. There is a paucity of literature regarding the cardiac involvement in COVID-19 infection. It is important for clinicians treating patients with COVID-19 to keep in mind that the infection may affect multiple organ systems, not just the respiratory system. Our case illustrates a rare complication of COVID-19 infection, myopericarditis and cardiac tamponade, which presented a diagnostic and therapeutic challenge and illustrates the importance of a systemic approach to treating COVID-19 infection.
